# Systematic Review of Host-Mediated Activity of Miltefosine in Leishmaniasis through Immunomodulation

**DOI:** 10.1128/AAC.02507-18

**Published:** 2019-06-24

**Authors:** Semra Palić, Patrick Bhairosing, Jos H. Beijnen, Thomas P. C. Dorlo

**Affiliations:** a Department of Pharmacy & Pharmacology, The Netherlands Cancer Institute-Antoni van Leeuwenhoek Hospital, Amsterdam, The Netherlands; b Scientific Information Service, The Netherlands Cancer Institute, Amsterdam, The Netherlands

**Keywords:** immunity, immunomodulation, leishmaniasis, miltefosine, Th1

## Abstract

Host immune responses are pivotal for the successful treatment of the leishmaniases, a spectrum of infections caused by *Leishmania* parasites. Previous studies speculated that augmenting cytokines associated with a type 1 T-helper cell (Th1) response is necessary to combat severe forms of leishmaniasis, and it has been hypothesized that the antileishmanial drug miltefosine is capable of immunomodulation and induction of Th1 cytokines. A better understanding of the immunomodulatory effects of miltefosine is central to providing a rationale regarding synergistic mechanisms of activity to combine miltefosine optimally with other conventional and future antileishmanials that are currently under development. Therefore, a systematic literature search was performed to evaluate to what extent and how miltefosine influences the host Th1 response. Miltefosine’s effects observed in both a preclinical and a clinical context associated with immunomodulation in the treatment of leishmaniasis are evaluated in this review. A total of 27 studies were included in the analysis. Based on the current evidence, miltefosine is not only capable of inducing direct parasite killing but also of modulating the host immunity. Our findings suggest that miltefosine-induced activation of Th1 cytokines, particularly represented by increased gamma interferon (IFN-γ) and interleukin 12 (IL-12), is essential to prevail over the *Leishmania*-driven Th2 response. Differences in miltefosine-induced host-mediated effects between *in vitro*, *ex vivo,* animal model, and human studies are further discussed. All things considered, an effective treatment with miltefosine is acquired by enhanced functional Th1 cytokine responses and may further be enhanced in combination with immunostimulatory agents.

## Abstract (precis)

Host immune responses are pivotal for the successful treatment of the leishmaniases, a spectrum of infections caused by *Leishmania* parasites. Previous studies speculated that augmenting cytokines associated with a type 1 T-helper cell (Th1) response is necessary to combat severe forms of leishmaniasis, and it has been hypothesized that the antileishmanial drug miltefosine is capable of immunomodulation and induction of Th1 cytokines.

## INTRODUCTION

With an estimated 678,000 cases and about 40,000 fatalities per annum worldwide, the neglected tropical disease leishmaniasis is the second largest parasitic killer, following malaria (1). Heterogeneity among parasite species results in different clinical manifestations, with visceral leishmaniasis (VL) being the most severe and potentially lethal form of *Leishmania* infection. In VL, parasites replicate within mononuclear phagocytic cells, leading to infection of the spleen, liver, and bone marrow (2, 3). Other clinical phenotypes include cutaneous (CL), post-kala-azar dermal (PKDL), and mucocutaneous (MCL) leishmaniasis, which are manifested by skin or mucous membrane lesions and/or ulcers (3, 4).

T-helper (Th) cells are the core of adaptive immunity, as their activity underpins almost every adaptive immune response, while impairments to Th cell functioning are found in many autoimmune diseases (5). When activated, naive Th cells divide and commit to a particular effector phenotype, including Th type 1 (Th1) or 2 (Th2). Th1 cells secrete cytokines such as alpha interferon (IFN-α), IFN-β, IFN-γ, interleukin-1β (IL-1β), IL-6, IL-8, IL-12, IL-18, IL-27, intercellular adhesion molecule 1 (ICAM1), tumor necrosis factor alpha (TNF-α), and TNF-β. Th1-related cytokines are particularly implicated in clearing intracellular pathogens, such as *Leishmania* parasites, that invade and replicate within reticuloendothelial cells (6, 7). Th2 cells primarily secrete IL-4, -5, -10, and -13, cytokines that activate pathways which are implicated in clearing extracellular pathogens and the development of allergies. The crucial role of Th1 activation in the treatment of VL has been demonstrated previously by the role of IFN-γ in infection clearance; e.g., IFN-γ knockout mice failed to respond to an anti-IL-4 monoclonal antibody treatment, resulting in progressive infection (8). Additionally, in mice with genetically compromised Th1 cytokine production, a dominant Th2 response led to exacerbated VL infection, implying that an active Th1 response is crucial in balancing the infection-promoting Th2 response and ultimately in controlling the parasite burden (9).

Although the Th1 versus Th2 dichotomy is typically less clear in human infection, the importance of the Th1/Th2 balance in obtaining control over the *Leishmania* infection has also been observed in clinical studies. Patients suffering from progressive VL showed a consistent lack of Th1 cytokine production (10), whereas expression of Th2 markers was detected in PKDL lesional tissues (10, 11). In VL and diffuse CL, an increase in Th2 activity is generally associated with infection progression, and Th1 activity has been associated with infection clearance and establishing clinical cure (7). IFN-γ has been shown effective as an adjunct therapy in VL and diffuse CL (12–15). However, in localized CL lesions, the balance between Th1 and Th2 cytokines appears more complex, since even the healing lesions were shown to contain persisting levels of circulating Th2 cytokines (16).

Due to the decreasing and disappointing efficacy of various antileishmanial drugs as monotherapy, including miltefosine, implementation of combination therapies is warranted. This is especially needed in East Africa, where antileishmanial drugs show lower efficacy rates systemically (17). To use new and existing therapies in the most optimal synergistic way, more knowledge on the direct and indirect mechanisms of action of antileishmanial compounds is required, e.g., through stimulation of the host immune system (18, 19). Miltefosine is an alkylphosphocholine agent (20), which is currently the only oral drug available on the market for the treatment of leishmaniasis and is widely used in the treatment of both CL and VL (21). Various direct and indirect antileishmanial mechanisms of action have been suggested for miltefosine, including disruption of (membrane) lipid metabolism, apoptosis-like cell death, induction of mitochondrial dysfunction, and also immunomodulatory effects involving Th1 cell response (20). Following the observation of the effects on Th cells in leishmaniasis, miltefosine has also been investigated in the treatment of other immune-mediated diseases, such as cancer, inflammatory bowel disease, and atopic dermatitis, showing promising preclinical results (22). Given the observed relationship between Th1/Th2 balance and control over the *Leishmania* infection, the potentiation of Th cell activation through the use of immunomodulators in addition to conventional chemotherapy has been hypothesized as a future therapeutic option for leishmaniasis (23, 24). Preclinical studies have, e.g., suggested that adding Th1-directed immunotherapy to chemotherapy could decrease *Leishmania*-associated suppression of the immune system and result in a more rapid parasite clearance (25–27). There is limited knowledge, however, about the translational and predictive value of immune effects from *in vitro* and various animal models for humans, which is complicated by intrinsic immunopathological differences between available murine and hamster models (28).

A better understanding of the immunomodulatory effects of miltefosine is central in providing a rationale regarding synergistic mechanisms of activity to combine miltefosine optimally with other conventional and future antileishmanials that are currently under development and immunotherapeutic interventions. Also, in principle, this may aid the understanding of the translational value of the immunomodulatory effects observed in preclinical models for other antileishmanial drugs. Therefore, our objective was to systematically review how miltefosine affects markers of the host Th1 response associated with its immunomodulatory effect in the treatment of leishmaniasis *in vitro*, in animal models, and in human and to what extent the host-mediated effects in these different models are in congruence.

## RESULTS

A complete search on 29 September 2017 yielded 56 hits in PubMed (MEDLINE), 94 hits in Embase (OVID), and 132 hits in Scopus. This search was repeated on 15 January 2018, resulting in 9 new hits, and again on 21 November 2018, resulting in 8 more new hits. After removal of the duplicates, 184 unique articles were identified in total.

**FIG 1:**
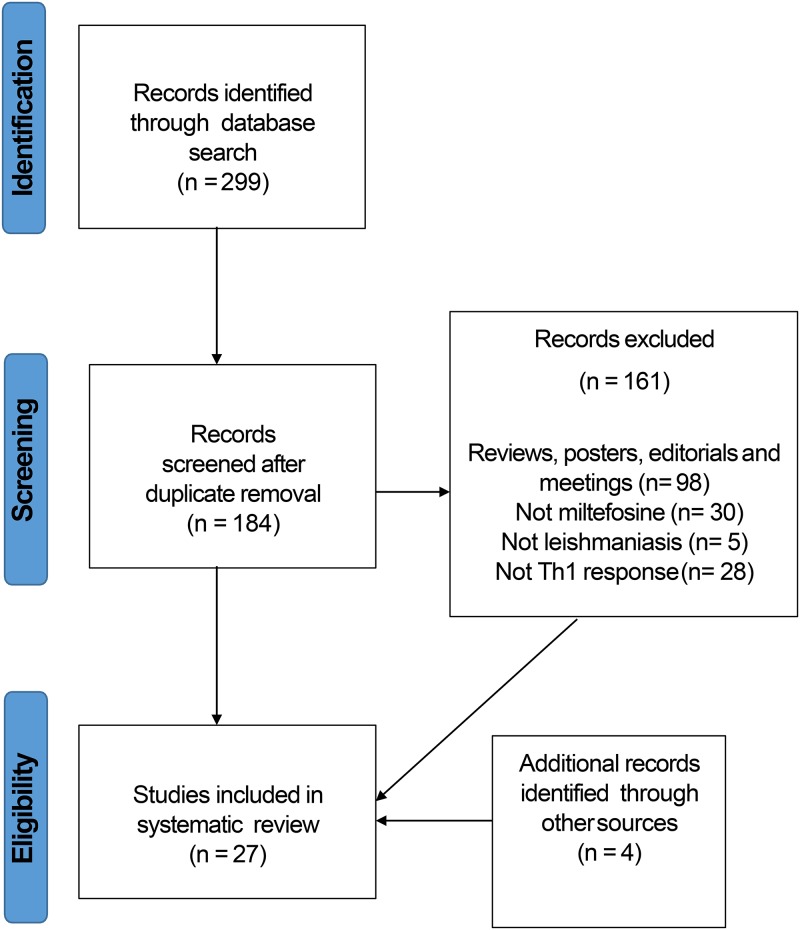
Flow chart of the studies identified, screened, and included in this review.

Our search identified in total 6 *in vitro*, 3 *ex vivo*, 13 animal, and 5 human studies which investigated Th1 cytokine activity after miltefosine treatment of leishmaniasis (Fig. 1). In total, 4 *in vitro* studies were available for VL (Leishmania donovani) and 1 for CL (Leishmania braziliensis and Leishmania major), as well as 2 *ex vivo* studies for VL (L. donovani) and 1 for CL (L. braziliensis). In animal studies, 12 identified studies investigated miltefosine effects in VL and 1 in CL, while in human studies, 3 studies were identified for VL and 2 for PKDL. Finally, a total of 27 studies were included in this systematic review: 23 meeting the inclusion and eligibility criteria from the primary search results and 4 studies identified through secondary sources. Results from all these studies in the various leishmaniasis disease models are discussed below.

### Studies *in vitro* and *ex vivo*.

*In vitro* studies (Table 1) were the first to propose and demonstrate that miltefosine induces a Th1 response via various immunological pathways. Applying miltefosine to *Leishmania*-infected splenocytes resulted in an induction of a Th1 response shown primarily by increased IFN-γ (19, 29–31). IFN-γ enables class switching from immunoglobulin G1 (IgG1) to IgG2. Analysis of IgG isotypes elucidated that IgG1 and IgG3 are significantly higher in patients with active VL, as well as in active lesions in CL, than control reference values in areas of endemicity (32, 33). IgG1 was proposed as a marker of relapse in Indian VL (32). Still, whether certain subclasses of IgG antibodies, such as IgG2, may have a protective role in VL and PKDL has not been clearly established. On the other hand, the levels of present antibody subtypes may illustrate the level of activation of cellular Th response (16). For instance, prior vaccination of mouse to increase IgG2 levels was associated with a 5-fold-higher IFN-γ level posttreatment (24). Furthermore, IL-12 and IFN-γ were significantly increased in miltefosine-treated cells *ex vivo* (Table 2), suggesting miltefosine-driven stimulation of Th1 cytokines (34). One mechanism proposed for the observed miltefosine immunomodulatory effects was the inhibition of phosphatidylinositol 3-kinase (PI3K)-dependent phosphorylation in macrophages and the simultaneous increase in the protein kinase C dependence, which in turn triggers the production of Th1 cytokines (30, 35). Another proposed mechanism of IFN-γ induction by miltefosine is that miltefosine may increase the expression of IFN-γ receptor, which further promotes signal transducer and activator of transcription 1 (STAT-1) signaling. In VL, STAT-1 phosphorylation is impaired by *Leishmania*-driven sphingosine-1-phosphate (SPH-1) activity. Miltefosine-mediated increases in IFN-γ responsiveness cause a decrease in SPH-1 activation, which in turn also leads to an increase in STAT-1 phosphorylation (30).

**TABLE 1:** Overview of *in vitro* studies of Th1 cytokine response following miltefosine treatment of leishmaniasis^*a*^

Study	Clinical indication	Parasite species	Host cell	Cytokine(s) measured (extracellular)	Direction of effects	No. of subjects (no. of groups)	Miltefosine concn(s) (μM)/dose	No. of time points evaluated	Time(s) between sampling intervals (h)
Gangalum et al. (29)	VL	L. donovani	Peritoneal macrophages (mice)	IFN-γ	Increase	5 (3)	0.8	1	24
Wadhone et al. (30)	VL	L. donovani	Peritoneal macrophages (mice)	IFN-γ, IL-12	Increase	5 (3)	3.2	5	1, 3, 6, 12, 24
Shukla et al. (31)	CL	L. major	Lymph node mononuclear cells	IFN-γ	Increase	8 (8)	NA	2	6
Ghosh et al. (19)	NA	NA	Peritoneal macrophages (mice)	IFN-γ, TNF-α	Increase	5 (5)	20	2	24, 72
Shivahare et al. (40)	VL	L. donovani	Macrophages (J-774A.1 cells) (mice)	IL-12, TNF-α	Increase	NA (6)	2, 8	1	48
Shivahare et al. (39)	VL	L. donovani	Macrophages (J-774A.1 cells) (mice)	IL-12, TNF-α	Increase	NA (5)	2, 8	1	48

a CL, cutaneous leishmaniasis; IFN, interferon; IL, interleukin; VL, visceral leishmaniasis; NA, not available.

**TABLE 2:** Overview of *ex vivo* studies of Th1 cytokine response following miltefosine treatment of leishmaniasis^*a*^

Study	Clinical indication	Parasite species	Host cell(s)	Cytokines measured	Direction of effects	No. of subjects (no. of groups)	Miltefosine concn(s)/dose	No. of time points evaluated	Time(s) between sampling intervals
Joshi et al. (38)	VL	L. donovani	Lymph node mononuclear cells (hamster), PBMCs (human)	IFN-γ, IL-12	Increase	30 animals, 32 humans (4)	Animals, 40 mg/kg; humans, NA	2	45 and 90 days
Mukherjee. et al. (34)	VL	L. donovani	THP1-derived macrophages	IFN-γ, IL-12, TNF-α	Increase	10	5 μM	2	24 and 48 h
Gonzalez-Fajardo et al. (37)	CL	L. braziliensis	PMBCs (human)^*b*^	IFN-γ, TNF-α, IL-12	Increase	22	2, 4, 8, 16, 32 μM	6	6, 12, 24, 48, and 72 h

a CL, cutaneous leishmaniasis; IFN, interferon; IL, interleukin; PBMCs, peripheral blood mononuclear cells; VL, visceral leishmaniasis.

b Monocyte-derived macrophages from isolated PBMCs.

In addition, infections with L. major and L. donovani are known to suppress the activation of p38 mitogen-activated protein kinases (p38MAPK), which is required for the production of proinflammatory Th1 cytokines (36). Miltefosine was able to increase the levels of p38MAPK activation in BALB/c-derived peritoneal macrophages, which in turn increased IL-12 levels in a dose-dependent manner within 48 h posttreatment (19, 29, 30). Moreover, miltefosine treatment of isolated peripheral blood mononuclear cells (PBMCs) derived from monocytes of VL patients resulted in an 8-fold rise in IL-12 levels (37). Also, PBMCs isolated from patients with advanced VL showed increased Th1 cytokines after *ex vivo* miltefosine treatment, further illustrating miltefosine-driven immunomodulation (38). Additionally, a functional role of miltefosine in the synthesis of TNF-α has been shown *in vitro* (29, 39, 40). BALB/c mouse-derived macrophages with a knockout platelet aggregation factor (PAF) receptor function displayed a complete lack of response to miltefosine, indicated by diminished miltefosine-induced parasite killing (29). Downregulation of the PAF receptor was also found to enhance IL-4 production, and suppress IFN-γ levels, resulting in progressive VL infection (29). Miltefosine is a structural analogue of PAF, and it was found that miltefosine activation of the PAF receptor led to increased IL-12 and TNF-α, while no effect was observed for PAF receptor-deficient macrophages (29, 41). Similar results were obtained based on CL patient-derived PBMCs (37).

### Studies in animals.

Various animal studies (Table 3) focused on the effects of miltefosine on IFN-γ production in both murine and hamster models of VL (42–49). Following miltefosine administration, all studies reported substantially increased IFN-γ levels in macrophages of *Leishmania*-infected animals in contrast to control groups (42–49). Several studies also showed that increases in IFN-γ levels were proportional to the dose of miltefosine administered and were accompanied by suppression of Th2-associated cytokine levels, together inducing the killing of parasites (43, 45, 50). As indicated by *in vitro* studies, IFN-γ activates macrophages, and miltefosine was shown to enhance the expression of IFN-γ in macrophages of BALB/c mice infected with L. donovani (42–44) Moreover, miltefosine was even able to upregulate IFN-γ levels in T-cell-deficient mice (46). In addition, control groups of mice and hamsters which were not treated with miltefosine demonstrated increased or unchanged parasite levels in comparison to those treated with subcurative and curative doses of miltefosine (50). Up to a 9-fold increase in Th1 cytokines was measured in mouse splenocytes 4 days posttreatment with miltefosine alone, which was boosted to a 13-fold increase when miltefosine was combined with immunostimulatory compounds such as pyrazolopyridine derivatives (45). These results indicate that the immune response shift toward Th1 is likely due to treatment-induced immunomodulation (51, 52).

**TABLE 3:** Overview of animal studies of Th1 cytokine response following miltefosine treatment of leishmaniasis^*a*^

Study	Clinical indication	Parasite species	Host	Measured cytokine(s)	Matrix	Direction of effects	No. of subjects (no. of treatment groups)	Miltefosine dose	Route of administration	Treatment duration	No. of time points per individual
Shakya et al. (42)	VL	L. donovani	Mouse	IFN-γ, IL-12, TNF-α	Serum	Increase	5–6 (9)	1.25 to 20 mg/kg q.d.	p.o.	5 days	2
Shakya et al. (43)	VL	L. donovani	Mouse	IFN-γ, TNF-α	Serum	Increase	5–6 (7)	2.5, 5, and 20 mg/kg q.d.	p.o.	7 days	2
Wege et al. (44)	VL	L. donovani	Mouse	IFN-γ	Spleen	Increase	32	2.5 mg/kg q.d.	p.o.	NA	3
Anand et al. (45)	VL	L. donovani	Mouse	IFN-γ, IL-12, TNF-α	Splenocytes	Increase	5 (7)	5 and 25 mg/kg q.d.	i.p.	5 days	2
Murray et al. (46)	VL	L. donovani	Mouse	IFN-γ	Peripheral blood	Increase	5 (8)	25 mg/kg q.d.	p.o.	5 days	3
Schmidt-Ott et al. (47)	CL	L. mexicana	Mouse	IFN-γ	Lymph nodes	Increase	6	1.5 mg q.d.	Topical	5 days/week for 5 weeks	2
Shivahare et al. (50)	VL	L. donovani	Mouse, hamster	IFN-γ, IL-12, TNF-α	Splenocytes	Increase	5–6 (9)	5 and 20 mg/kg q.d. (mouse), 5 and 40 mg/kg q.d. (hamster)	p.o.	5 days	2
Sane et al. (53)	VL	L. donovani	Mouse, hamster	IFN-γ, TNF-α	Serum	Increase	5 (6)	2.5 to 40 mg/kg q.d.	p.o.	5 days	2
Gupta et al. (49)	VL	L. donovani	Hamster	IFN-γ, IL-12, TNF-α	peripheral blood	Increase	8–10 (5)	40 mg/kg q.d.	NA	5 days	2
Jaiswal et al. (48)	VL	L. donovani	Hamster	IFN-γ, IL-12	peripheral blood	Increase	30	40 mg/kg q.d.	p.o.	5 days	3
Tripathi et al. (51)	VL	L. donovani	Hamster	IFN-γ, IL-12	Lymphocytes	Increase	5–6 (9)	2.5 to 40 mg/kg q.d.	p.o.	5 days	2
Manna et al. (55)	VL	L. infantum	Dog	IFN-γ	Peripheral blood	Increase	20	2 mg/kg q.d.	p.o.	30 days	5
Andrade et al. (52)	VL	L. chagasi	Dog	IFN-γ	Peripheral blood	Increase	14	100, 200 mg/animal q.d.	NA	28 or 45 days	3

a CL, cutaneous leishmaniasis; IFN, interferon; IL, interleukin; i.p., intraperitoneal; NA, not available; PKDL, post-kala-azar dermal leishmaniasis; p.o., per os; q.d., once daily; VL, visceral leishmaniasis.

**FIG 2:**
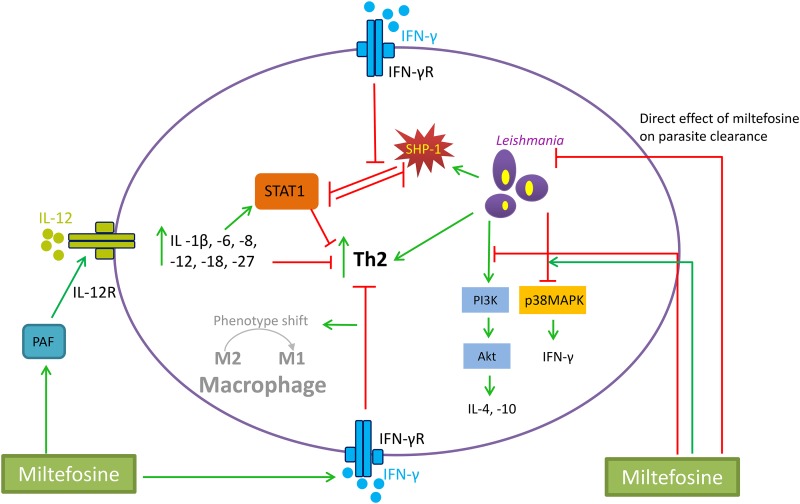
Proposed mechanisms of action for miltefosine. The proposed mechanisms include direct killing of *Leishmania* parasites and several immunomodulatory effects, which are exerted via (i) platelet aggregation factor (PAF) receptor, increasing production of interleukin (IL)-12, (ii) enhancement of interferon gamma (IFN-γ) receptor, which in turn lowers the production of T-helper (Th) cell type 2 cytokines (such as IL-4, -5, -10, and -13), (iii) activation of IFN-γ, reversing sphingosine-1-phosphate (SPH-1) inhibition of signal transducer and activator of transcription 1 (STAT-1), which is translocated to the nucleus and involved in stimulation of the host cellular immunity, (iv) activation of p38 mitogen-activated protein kinase (p38MAPK), which is initially inhibited by *Leishmania*, and (v) inhibition of PI3 kinase phosphorylation of protein kinase B (Akt), which is initially stimulated by the parasite. Red lines indicate an inhibitory effect, while green arrows indicate a stimulatory effect.

Studies in mice have also documented a dose-proportional increase in IL-12 after standard miltefosine treatment. The host immune system requires IL-12 in order to stimulate the differentiation of Th1 cells, further maintain Th1 responses, and overall stimulate the production of IFN-γ (42, 43). A few studies argued that miltefosine-mediated immunomodulatory effects are more advanced when miltefosine is administered in combination with compounds that stimulate Th1 polarized cytokines (50, 53). As already postulated by *in vitro* studies, an increase in IgG2 antibodies contributed to the development of an effective Th1-mediated immune response (45, 51, 53). Increases in IgG2 expression, as well as consequent suppression of IgG1 dominance, were more appropriately illustrated in hamster models, since mice lack the distinct subclasses among IgG antibodies (54). In hamster VL (L. donovani) models, upon successful miltefosine treatment, complete cure is reached at day 45, where IFN-γ, IL-12, and TNF-α levels were identified as indicators of treatment outcome (48, 49). Furthermore, two studies evaluated the effects of miltefosine in the treatment of dogs naturally infected with VL (L. infantum). A 28- or 45-day treatment with oral miltefosine administered daily (100 to 200 mg/day) increased IFN-γ in peripheral blood up to 2-fold at day 180 after start of treatment (52). Subsequent relapse in these dogs was associated with decreased IFN-γ and reoccurrence of Th2 cytokine production. Relapses were primarily associated with increases in IL-4 and IL-10, which is typically observed at the time of diagnosis (52). Dogs treated with a combination of miltefosine and allopurinol showed a more prolonged increase of IFN-γ in peripheral blood, with 90% survival at 9 months (55). Lastly, a single study investigating the immune effects of miltefosine on CL in BALB/c, CBA/J, and C57BL/6 mouse models infected with either L. major or Leishmania mexicana showed that IFN-γ was elevated in lymph nodes directly during and after 5 weeks of miltefosine treatment. For L. major infection in BALB/c mice, the most significant increase in IFN-γ was 3.1-fold at 3 weeks after the 5-week miltefosine treatment, while the increases in IFN-γ for CBA/J and C57BL/6 mice were 2.8- and 1.9-fold, respectively (47). Differences in IFN-γ receptor expression were observed among the various murine species. Miltefosine treatment of L. mexicana infection in mice was ineffective and did not result in increased levels of Th1 cytokines, resulting in 9 out of 12 disease relapses (47). However, a relatively low miltefosine dose was administered, and thus an optimal exposure might not have been achieved (47). Taken together, preclinical studies show the importance of immune cross talk in both infection development and clearance. Miltefosine immunomodulation in the animal models appears to be exerted through two major pathways (Fig. 2): stimulation of Th1 cytokine production that will further drive macrophage activation and activation of the transcription factors within the infected macrophages, which will in addition prime macrophages to increase Th1 cytokine secretion and eventually counterbalance anti-inflammatory cytokines.

### Studies in human.

Although very limited results from studies in human (Table 4) are available to date, these appear to be in line with findings from *in vitro* and animal studies. Production levels of IFN-γ and IL-12 were also found elevated after miltefosine treatment of VL (56). Additionally, in patients with inborn errors in the genes encoding IL-12, miltefosine was shown ineffective and VL reoccurred multiple times (57). These patients were also more susceptible to other infections, such as tuberculosis. This indicates that impaired IFN-γ functioning does not only hamper normal immune function but also limits miltefosine’s action to stimulate macrophage-driven Th1 cytokine production. The importance of restoring the host immunity in VL is particularly demonstrated in immunosuppressed patients, such as those who are coinfected with human immunodeficiency virus (HIV), where VL is even more challenging to treat and results in more episodes of relapse and even death (58). Furthermore, neopterin, a Th1 immune marker, has been recently evaluated in VL patients after treatment with miltefosine alone or in combination with amphotericin B (59). Neopterin is exclusively produced by macrophages, which are activated by IFN-ɣ upon treatment (60, 61). As such, a decline in neopterin levels due to miltefosine treatment may directly reflect a decline in macrophages loaded with parasites.

**TABLE 4:** Overview of human studies of Th1 cytokine response following miltefosine treatment of leishmaniasis^*a*^

Study	Clinical indication	Parasite species	Measured cytokine(s)	Matrix	Direction of effects	No. of subjects (no of treatment groups)	Miltefosine dose	Route of administration	Treatment duration	No. of time points
Das et al. (56)	VL	L. donovani	IFN-γ, TNF-α	Peripheral blood	Increase	23	50 mg b.i.d.	p.o.	28 days	2
Parvaneh et al. (57)	VL	L. infantum	IFN-γ, IL-12	Whole blood	Increase	2	NA	p.o.	28 days	NA
Kip et al. (59)	VL	L. donovani	Neopterin	Plasma	Decrease	48 (2)	2.5 mg/kg/day	p.o.	28 days, 10 days	6–7
Ansari et al. (10)	PKDL	L. donovani	IFN-γ, TNF-α	Lesional tissue biopsy specimen	IFN-γ increase, TNF-α decrease	1	50 mg b.i.d. or t.i.d.	p.o.	15 weeks	3
Mukhopadhyay et al. (62)	PKDL	L. donovani	TNF-α, IL-1β, IL-6, IL-8	Peripheral blood	Increase	16	100 mg q.d.	p.o.	16 weeks	2

a b.i.d., twice daily; CL, cutaneous leishmaniasis; IFN, interferon; IL, interleukin; i.p.: intraperitoneal; NA, not available; PKDL, post-kala-azar dermal leishmaniasis; p.o., per os; q.d., once daily; t.i.d., three times daily; VL, visceral leishmaniasis.

Additionally, somewhat different immunological responses were observed in PKDL, where both Th1 and Th2 cytokines were found to be present. Since PKDL patients have already been treated for VL, infection is no longer systemic, as the result of treatment-associated increased Th1 cytokine levels. However, Th2 cytokines are still present in the skin, most likely remaining since the primary VL infection (11). One of the case studies identified describes a decrease of IFN-γ in a patient’s lesional tissue after treatment with miltefosine (10). Measured CD40 levels were also found enhanced and probably contributed to the evoked Th1 signaling. However, TNF-α levels were found decreased, which may be explained by the concomitant treatment of rheumatoid arthritis with the immunosuppressant hydroxychloroquine and by previous mistreatment of leprosy with clofazimine, both known to interact with Th signaling (10). In addition, Mukhopadhyay et al. (62) reported that miltefosine significantly increased the secretion of Th1 cytokines and decreased the anti-inflammatory Th2 responses in PKDL patients. As with scenarios observed in cases of VL, elevated levels of TNF-α, IL-1β, IL-6, and IL-8 in peripheral blood were also accompanied by higher levels of serum nitrate, which are known to drive proinflammatory monocyte responses in PKDL (62). Development of PKDL has been reported after treatment of VL with various antileishmanials, including miltefosine, amphotericin B, stibogluconate, and paromomycin, but the rates of occurrence of PKDL after any of the treatments have not been studied to date (63, 64). Understanding immunological responses during VL treatment is therefore of crucial importance for advancing our knowledge about infection reappearance in some patients during asymptomatic intervals.

## DISCUSSION

In the current review, we have systematically evaluated and summarized the proposed immunomodulatory effects in the treatment of various leishmanial infections *in vitro*, *ex vivo*, in animal, and in human. To the best of our knowledge, this is the first systematic review of the host-mediated activity of miltefosine through immunomodulation. Several general mechanisms were identified to support miltefosine-mediated immunomodulation. *Leishmania* parasites drive the Th2 response during the course of infection in VL, and miltefosine was found capable of reversing these infection-driven effects, especially demonstrated in VL subjects (49, 65). *Leishmania* reduces the responsiveness of IFN-γ receptors within infected cells, while miltefosine has been found to restore the functioning of IFN-γ receptors (66). In having such a direct effect on IFN-γ receptors, miltefosine is able to activate a proinflammatory immune response, along with parasite killing (67, 68). IFN-γ alone appears insufficient to drive a dominant Th1 response; IL-12 also has an important role in sustaining this Th1 response (51, 69). *In vivo* studies showed that miltefosine induced IL-12 in a dose-dependent manner (30). Animal studies further demonstrated that a complete cure at the end of the treatment was associated with a rise in IFN-γ, IL-12, and TNF-α levels, suggesting that these cytokines may indicate an initial treatment response (48, 49). Essentially, increased concentrations of proinflammatory cytokines will lead the shift in macrophage phenotype from M2, which is dominated by Th2 cytokine expression, to M1, which is driven by Th1 cytokines and ultimately needed to clear the intracellular pathogen. Several studies further reported higher concentrations of Th1 cytokines when miltefosine was combined with compounds known to stimulate the host immunity. It has been long hypothesized that various antileishmanial drugs, including miltefosine, amphotericin B, paromomycin, and antimonials, exert immunomodulatory effects. However, to our knowledge, only a single *in vitro* study made a direct comparison between different antileishmanials and IL-12 levels, where it was reported that miltefosine, amphotericin B, and sodium antimony gluconate-treated macrophages produce increased IL-12 levels but not macrophages treated with paromomycin (19). It remains difficult to evaluate to what extent miltefosine immunomodulatory effects might be different from those of other antileishmanials, or whether observed effects result from decreasing parasite levels or rather direct effects on Th1 cell signaling. However, in contrast to other antileishmanials, immunomodulatory effects have been reported only for miltefosine in immune-mediated disorders such as rheumatoid arthritis, chronic urticaria, or malignant disease (22). Taken together, these findings illustrate that besides the direct killing of the parasite, miltefosine is also able to affect the host immune system by targeting Th1. These observations not only indicate the mechanisms of miltefosine immunomodulation but also highlight the importance of Th1 cytokine activation for the clearance of *Leishmania* in VL. Moreover, in spite of the fact that only a few human studies were identified in our review of the available literature, the studies that were identified support the role of Th1 cytokine activation by miltefosine in the treatment of VL. This is corroborated by IL-12-deficient VL patients in which miltefosine was nonefficacious. However, a contrasting change in levels of neopterin is observed upon miltefosine treatment in VL patients. While most Th1 cytokines reflect cascades taking place outside, or at the surface, of macrophages that are needed for its activation, neopterin reflects an activated macrophage response, whose decline therefore is attributed to a decline in macrophage parasite level.

As mentioned, the immunomodulatory effects of miltefosine have also been demonstrated in the treatment of other immune-mediated disorders, such as inflammatory bowel disease (IBD) and chronic urticaria (22). In a mouse model of IBD, miltefosine was shown to block the proliferation of Th2 cytokines, subsequently increasing Th1 cytokines, which resulted in the decline of inflammation and less severe colitis (70). Moreover, in patients with chronic spontaneous urticaria who do not respond to treatment with antihistamines, miltefosine was able to relieve symptoms such as the number of weals and the intensity of pruritus (71). Additionally, miltefosine-induced immunomodulation may particularly be important for patients who are coinfected with HIV. In this immunocompromised patient population suffering from both HIV and VL, the immune responses are heavily dominated by Th2 cytokine activity, and combined with antiretroviral treatment, miltefosine was proven to have relatively high efficacy (58, 72).

Taken collectively, the identified *in vitro*, *ex vivo*, animal, and human studies suggest the therapeutic importance of miltefosine-driven activation of Th1 cytokines in the treatment of VL, diffuse CL, and PKDL (30, 45, 73). A direct translation between the various test systems and the corresponding therapeutic effects nevertheless may not be easily derived. Underlying reasons are the complex physiological and immunological factors that are known to result in different pathophysiologies and immunopathologies between species (74). For example, untreated VL in hamster models typically results in mortality, while in murine models, the parasite is cleared and infected subjects recover even in the absence of treatment (75). Moreover, murine VL models usually reflect acute infections in spleen and liver that may be resolved, and parasite clearance from these organs does not necessarily represent immune sterilization (23). This is contrast to human infection, where the stage of leishmanial infection may not be obvious and patients may suffer from concomitant diseases or infections that may also compromise the immune system (76). In addition, some immunological aspects observed in human patients could not be accurately reproduced in mice. For example, glucocorticoid-induced tumor necrosis factor receptor family-related protein (GITR) was found increased in VL patients. Pharmacological blockage of its receptor did not induce antiparasitic immunity and even restored IL-10 levels that were initially inhibited by IFN-γ (77, 78). In mice, however, due to contrasting humoral and cellular immune responses, these effects were not observed (75, 77).

Furthermore, differences in activation between Th1 and Th2 cytokines appear more obvious in mice; therefore, the initial distinction between these cytokines has been derived based on studies in mice. However, studies in human showed that a strict distinction between Th1 and Th2 cytokines is too simplistic, as both the disease and the treatment will drive the balance between these cytokines through inhibitory and positive feedback loops (7, 79). In PKDL specifically, Th17 cytokines such as IL-17 and TNF-α are found upregulated compared to the control. Th17 cytokines are known to recruit neutrophils to induce tissue inflammation and link the innate to adaptive responses. Therefore, different leishmanial infections in human appear to drive distinct T cell differentiation, which suggests that immunomodulation between parasite species is also different (78, 79). In essence, *in vitro* studies provide crucial information on how the parasite and separate immune cells respond to the drug. However, these studies may often lack the power to illustrate how the complete host immune system may respond to treatment (63). Therefore, in order to translate findings between preclinical and clinical studies, we highlight the need of implementing translational pharmacokinetic-pharmacodynamic (PK-PD) modeling. PK-PD modeling and simulation have already shown superiority over classical extrapolation and translation of preclinical to clinical findings and have demonstrated particular benefits in the development and evaluation of dosing regimens in special patient populations such as children or pregnant women (80, 81).

According to the identified studies, miltefosine is able to actively influence the host immunity through the stimulation of production of Th1 cytokines that participate in *Leishmania* clearance. In line with this, several preclinical studies also proposed that a combination treatment with immunostimulatory agents may enhance miltefosine’s immunomodulation and result in a more favorable treatment outcome, especially in cases where infections are advanced or where the immune system is further compromised by the presence of additional coinfections (39, 42, 50). In conclusion, while targeted immunotherapy in the treatment of VL is still lacking, given its modulatory effects, we emphasize the potential of miltefosine in synergy with future antileishmanial compounds.

## MATERIALS AND METHODS

### Search strategy.

A systematic search of the literature was performed in PubMed (MEDLINE), Embase (OVID), and Scopus on 29 September 2017 and repeated on 15 January 2018 and 21 November 2018. The sensitivity of the search was accomplished by including the following terms: “Miltefosine” AND “Leishmaniasis” AND (“Th1-cells” OR “Cytokines” OR “Chemokines” OR “Intercrines” OR “Interleukins”). All terms were searched in MeSH terms (or equivalent in other databases), title, and abstract. The full search strategies are shown in Data File SI in the supplemental material. Deduplication of the articles was done according to the method of Bramer et al. (82).

### Study identification and selection.

No limits were used in the search strategy. Inclusion and exclusion of found literature were performed in accordance with PRISMA guidelines (Data File SII) (83). Secondary sources were identified through the referenced literature of the primary identified studies and through additional querying of PubMed using the search terms “Miltefosine” AND (“Immunity” OR “Immunomodulation” OR “Immunomodulatory”). Language restrictions were not applied in the selection of studies. Studies with only titles or abstracts available were not included. Eligible studies had to describe the effects of miltefosine on Th1 activity, demonstrating a change in the levels of any of the following, since these markers have been associated with cellular immunity and subversion of intracellular pathogens: IFN-α, IFN-β, IFN -γ, IL-1, IL-6, IL-8, IL-12, IL-18, IL-27, ICAM1, neopterin, TNF-α, and TNF-β. Therefore, studies focusing on other immunological factors such as changes in Th2 cytokines and alterations in Toll-like receptor expression were not included. Only original research articles and treatment-relevant patient case reports (focused on Th1 response) were included. Therefore, reviews, editorials, commentaries, posters, conference reports, and nonimmunological case reports, etc., were excluded. We aimed to evaluate the effects observed in both a preclinical and clinical context; hence, we included all *in vitro*, animal, and human studies. The literature identified by the search strategy was screened independently by two authors (S.P. and T.P.C.D.) for the eligibility criteria mentioned above. Disagreements were resolved by discussion between the two authors.

### Data extraction.

The following information was retrieved and extracted from each included study: clinical presentation of the leishmaniasis infection (VL, CL, or PKDL), parasite subspecies, number of subjects, population age range, male-to-female ratio, and geographical region, which were applicable only to human studies, and miltefosine dose, route of administration, dosing frequency, treatment duration, sampling schedule, sampling matrix, measured cytokine(s), and direction of observed effects. Where applicable, we also included the dosing schedule of comedication, the presence of comorbid diseases or conditions, the follow-up period, and whether a correlation was observed between the immunological markers of interest and treatment outcomes, such as initial treatment response, relapse, or final cure.

## Supplementary Material

Supplemental file 1
